# A drug–drug cocrystal of breviscapine and borneol: harnessing natural product synergy for synchronized release and improved pharmacokinetics

**DOI:** 10.1007/s13659-026-00648-6

**Published:** 2026-11-02

**Authors:** Jiao Wang, Caixian Wang, Yan Ma, Tong Zhang, Anguo Hou, Wenbin Jin, Yanni Ma, Wenping Wang

**Affiliations:** 1 Yunnan Key Laboratory of Chinese Medicine Processing, College of Chinese Materia Medica, Yunnan University of Chinese Medicine, Kunming, 650500 China; 2 General Hospital of Ningxia Medical University, Yinchuan, 750004 China

**Keywords:** Drug–drug cocrystal, Supramolecular interaction, Natural products, Breviscapine, Borneol, Oral bioavailability

## Abstract

A drug–drug cocrystal strategy harnessing natural product synergy was developed to address the oral delivery barriers of breviscapine (BRE), a natural flavonoid with poor solubility and extensive first-pass metabolism. BRE was integrated with the monoterpenoid permeation enhancer borneol (BO) via solvent evaporation to form a stable supramolecular assembly. Comprehensive solid-state characterization and density functional theory calculations confirmed that the cocrystal lattice is stabilized by specific hydrogen bonding between the flavonoid and terpenoid scaffolds. The optimized BRE-BO cocrystal exhibited highly synchronized release of both natural products (*R*^2^ = 0.86), in contrast to the discordant dissolution of the physical mixture, with kinetics following the Korsmeyer–Peppas model (n = 0.45) indicative of Fickian diffusion. In vivo pharmacokinetic evaluation in rats demonstrated that the synergy conferred by co-delivered BO resulted in a 1.6-fold enhancement in oral bioavailability (*AUC*_0-24_), a prolonged *T*_max_ (from 2.3 to 3.3 h), and an elevated *C*_max_ (1.2 vs. 0.8 mg/L) compared to bulk BRE. These findings demonstrate that drug–drug cocrystallization can simultaneously achieve dissolution synchronization and pharmacokinetic optimization, providing a viable strategy for the formulation of poorly bioavailable natural flavonoids without the use of polymeric excipients.

**Graphical Abstract:**

**Figure:**
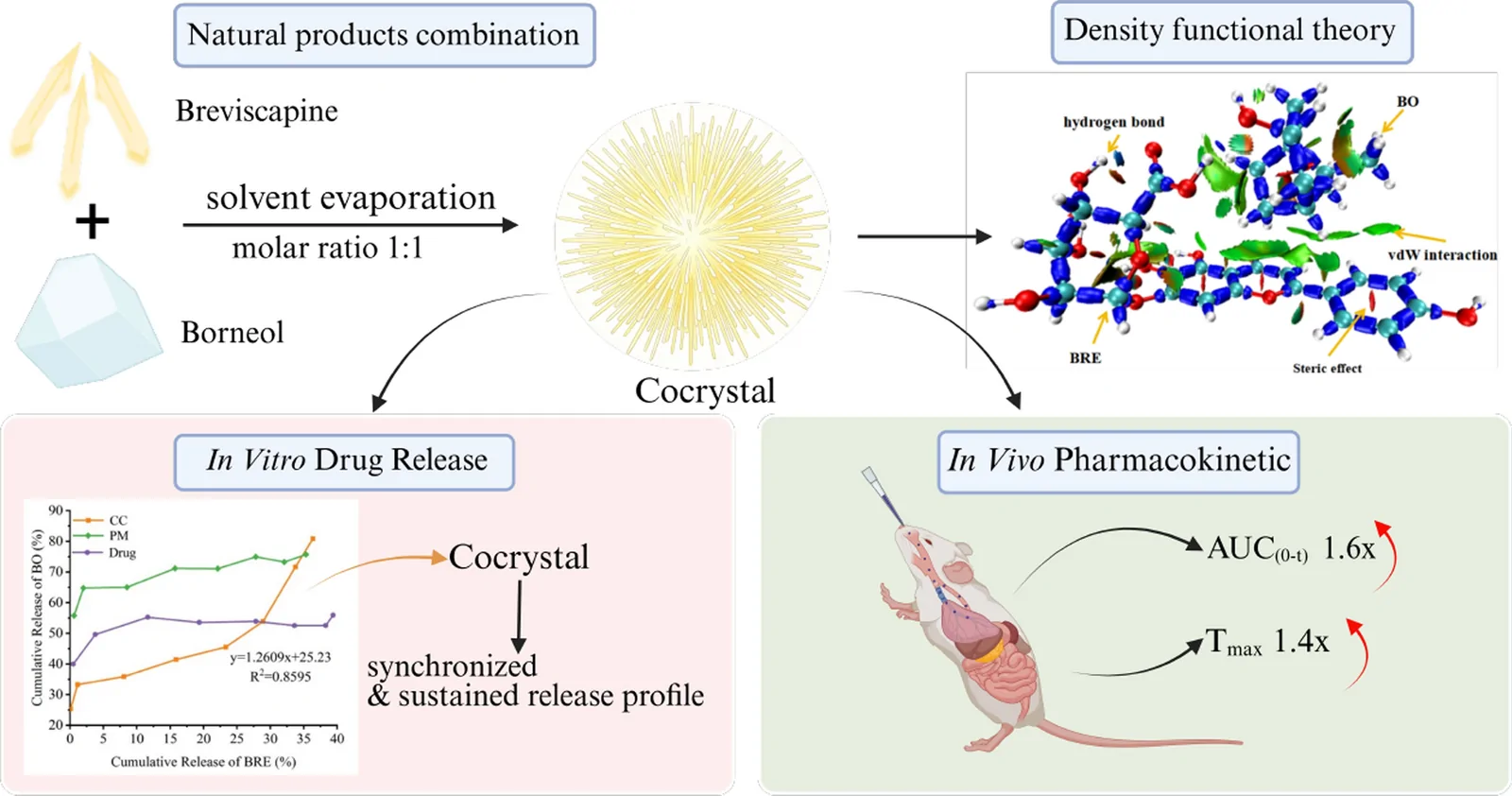

## Introduction

**Fig. 1:**
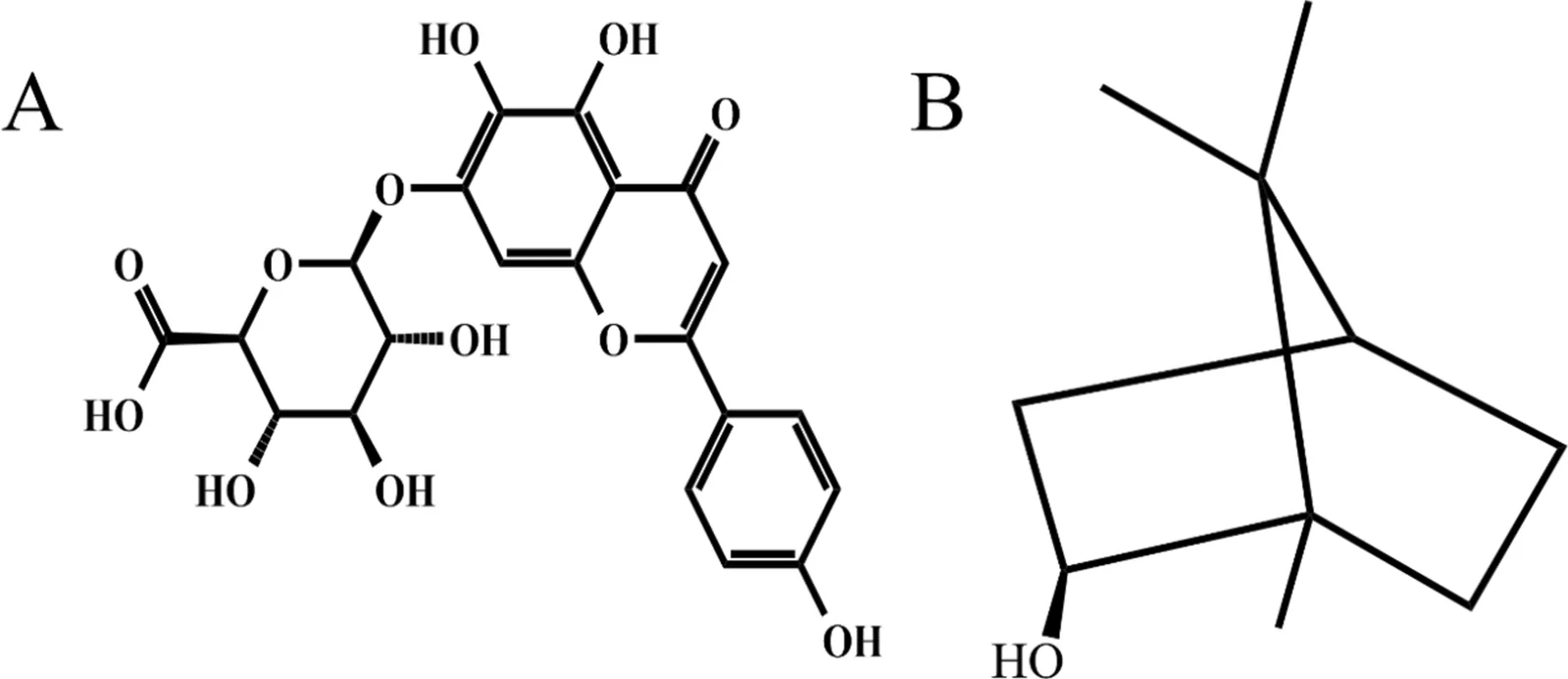
Chemical structure of **A** scutellarin and **B** borneol

Natural flavonoids derived from Traditional Chinese Medicine (TCM) represent a vast reservoir of bioactive compounds with therapeutic potential in cerebrovascular, cardiovascular, and inflammatory diseases [1]. Breviscapine (BRE), a standardized flavonoid extract from *Erigeron breviscapus* containing > 85% scutellarin (Fig. 1A), exemplifies both the promise and the limitations of this compound class. While scutellarin exhibits potent anti-inflammatory, antioxidant, and vasoprotective activities [2], its clinical utility is severely compromised by poor aqueous solubility and extensive first-pass glucuronidation, which collectively result in low and inconsistent oral bioavailability [3]. Although advanced formulation strategies—including nanoparticles, liposomes, and solid dispersions-have been employed to overcome these obstacles [4], their clinical translation is often impeded by low drug loading capacities, complex manufacturing processes, and the extensive use of synthetic excipients that may deviate from the “natural product” origin [5]. This challenge underscores the need for alternative, streamlined strategies that enhance biopharmaceutical performance while respecting the natural origin of the therapeutic agents.

Recent progress in crystal engineering has positioned pharmaceutical cocrystals as a compelling supramolecular solution to such biopharmaceutical challenges [6]. Distinct from conventional salts or amorphous solid dispersions, cocrystals facilitate the precise spatial integration of an active pharmaceutical ingredient (API) with a pharmaceutically acceptable coformer within a single crystalline lattice [7]. This molecular-level modulation enables the tailored optimization of physicochemical properties—such as solubility and intrinsic dissolution rate—without requiring covalent modification of the API’s pharmacophore [8]. Beyond mere solubility enhancement, there is mounting evidence that co-crystallization can be exploited to engineer sustained drug release profiles by modulating lattice energy and the associated dissolution-diffusion kinetics, thereby offering a dual-functional approach to oral delivery optimization [9,10].

In this context, Borneol (BO), a bicyclic monoterpene renowned in TCM as a “guide drug” (Yinjing agent) (Fig. 1B), presents a unique opportunity as a coformer with intrinsic therapeutic synergy [11]. Pharmacologically, BO is well-documented to enhance intestinal absorption by reversibly modulating tight junction integrity and inhibiting P-glycoprotein-mediated efflux and cytochrome P450-mediated metabolism [12,13]. Structurally, its rigid terpene skeleton features a hydroxyl group capable of engaging in specific hydrogen bonding interactions with the phenolic and hydroxyl moieties of flavonoid scaffolds [14]. Despite the recognized potential of natural product coformers [15], a systematic investigation into the development of a drug–drug cocrystal leveraging BO simultaneously as a structural modulator and a functional bio-enhancer for flavonoid delivery has not been reported. Herein, we report the rational design and characterization of a BRE-BO pharmaceutical cocrystal aimed at synchronizing dissolution kinetics and augmenting intestinal absorption of BRE. The supramolecular assembly mechanism was rigorously elucidated using a combination of thermal analysis, vibrational spectroscopy, solid-state nuclear magnetic resonance, and density functional theory (DFT) calculations. Furthermore, comparative in vitro dissolution and in vivo pharmacokinetic studies were performed to validate the translational potential of this excipient-free, crystalline formulation strategy.

## Results and discussion

### Morphological evolution and crystallization habit

**Fig. 2:**
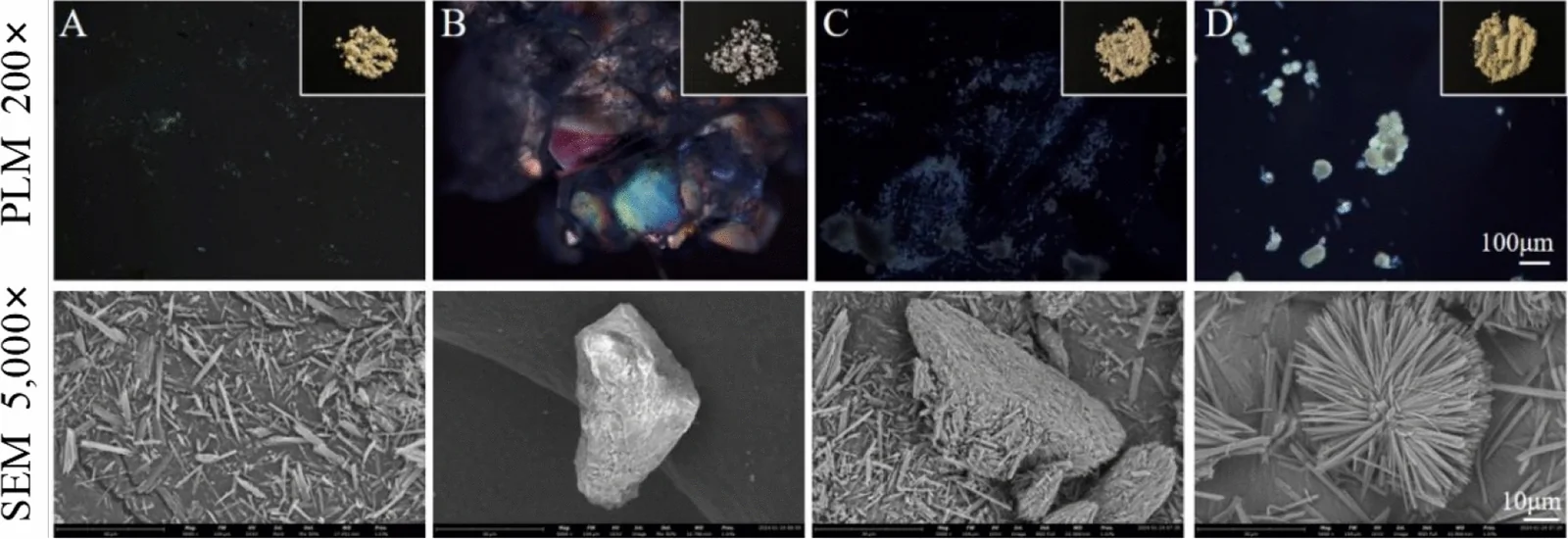
Comparative morphological analysis: **A** Pure BRE, **B** Pure BO, **C** BRE-BO physical mixture (PM, 1:1 molar ratio), and **D** BRE-BO cocrystals (CC). Insets: Macroscopic appearance of the respective powders

The transformation in crystal habit serves as a primary indicator of cocrystal formation. The microscopic morphology of BRE, BO, their PM and CC was systematically characterized via PLM and SEM, as illustrated in Fig. 2.

Pure BRE (Fig. 2A) exhibited characteristic agglomerates of irregular rod-like crystals (approx. 10–20 μm in length) with weak birefringence, consistent with previous reports on its crystalline nature [16]. In contrast, pure BO (Fig. 2B) presented as large, irregular polycrystalline aggregates (50–300 μm) displaying intense, multi-colored birefringence, indicative of its high crystallinity and optical anisotropy [17]. The physical mixture (Fig. 2C) appeared as a disordered combination of BRE rods mechanically interspersed with BO blocks, retaining the distinct morphological signatures of the individual components without evidence of interaction.

Crucially, the BRE-BO cocrystal (Fig. 2D) demonstrated a radical modification in crystal habit. The product crystallized into hierarchical, uniform spherulitic superstructures (40–50 μm in diameter). SEM analysis revealed that these spheres were composed of centripetally aggregated, fine rod-like subunits (20 ± 3 μm in length). Under PLM, these structures displayed a uniform and distinct birefringence pattern that differed from those of the parent materials. This morphological homogeneity suggests the formation of a single, pure crystalline phase via the co-evaporation process, ruling out a simple physical mix and indicating a lattice-level reorganization driven by specific intermolecular recognition between the flavonoid and terpenoid components.

The observed morphological uniformity of the cocrystal, distinct from the heterogeneous physical mixture, provides initial visual evidence for the successful formation of a drug–drug cocrystal phase. Furthermore, the hierarchical spherulitic architecture—comprising radially aligned rod-like subunits—may contribute to the modified dissolution kinetics described below, as the altered packing density and surface characteristics can modulate solvent penetration and lattice dissociation rates.

### Solid-state characterization and intermolecular interactions

**Fig. 3:**
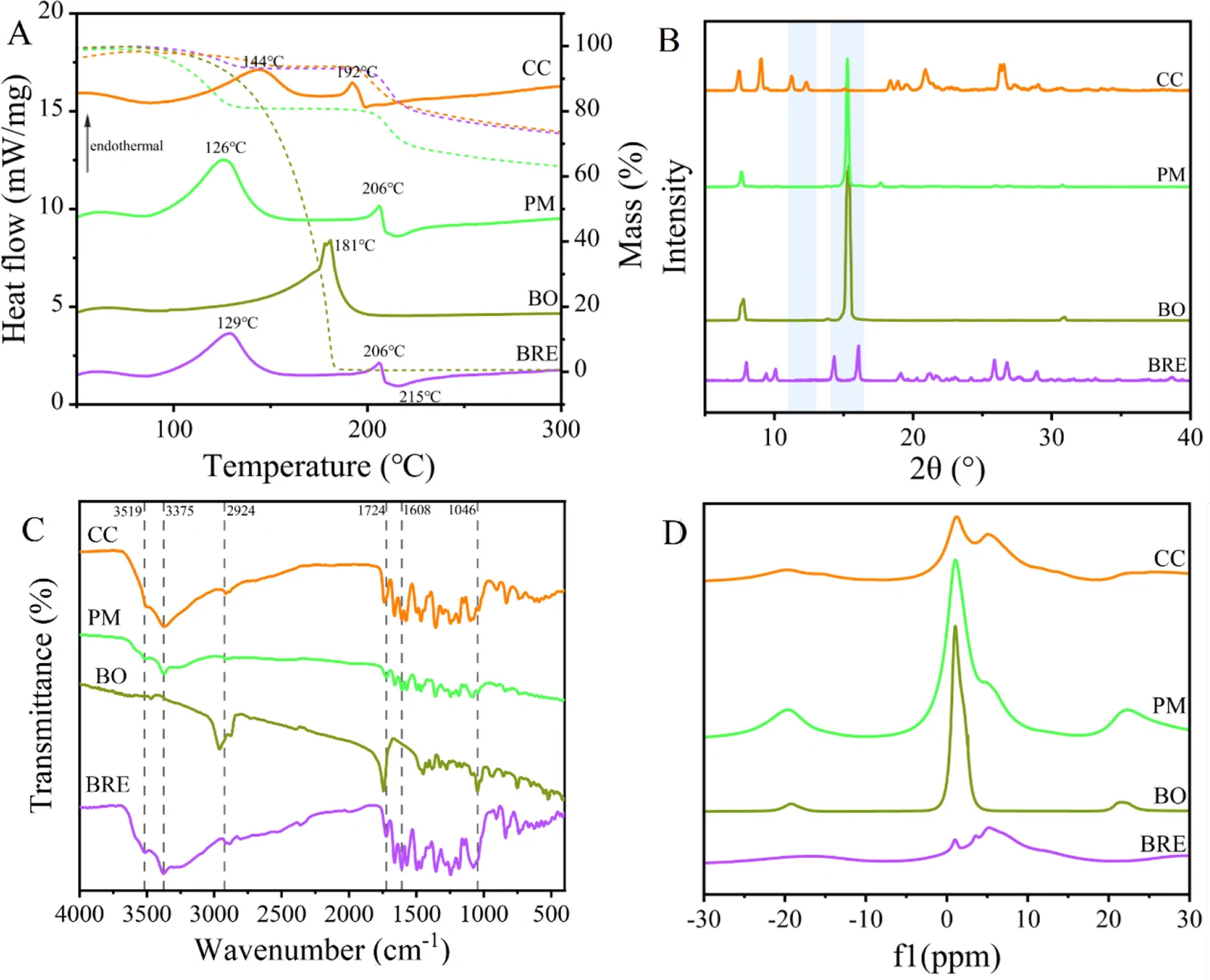
Multimodal characterization of BRE, BO, their physical mixture (PM) and cocrystal (CC): **A** DSC/DTG thermograms; **B** PXRD patterns; **C** FTIR spectra; **D** Solid-state ^1^H-NMR spectra

To confirm the formation of the cocrystal and elucidate the driving forces behind its assembly, a multimodal solid-state analysis was conducted (Fig. 3).

#### Thermal analysis

The thermal behavior of the samples (Fig. 3A) provided quantitative thermodynamic evidence of cocrystallization. Pure BRE exhibited two endothermic events: a dehydration peak at 129 °C (ΔH = 177.7 ± 3.2 J/g) and a melting peak at 206 °C (ΔH = 46.49 ± 1.8 J/g) [16]. BO displayed a sharp, single endotherm at 181 °C (ΔH = 210.4 ± 4.5 J/g), characteristic of volatile terpenes [18]. The thermogram of the PM was essentially a superposition of the individual components (BRE peaks at 129 °C and 206 °C; BO at 181 °C), indicating negligible interaction during physical mixing.

Conversely, the BRE-BO cocrystal revealed a unique thermal profile distinct from those of the starting materials. It exhibited a broad endotherm at 144 °C (ΔH = 153.2 ± 2.7 J/g) followed by a new melting event at 192 °C (ΔH = 38.6 ± 1.3 J/g). The complete disappearance of the characteristic BO melting peak (181 °C) and the shifts in BRE thermal transitions indicate that BO was successfully incorporated into the BRE crystal lattice, forming a new phase with altered lattice energy [19,20]. The intermediate melting endotherm observed for the cocrystal suggests a moderate lattice stabilization that may facilitate gradual dissolution under physiological conditions, rather than the rapid sublimation characteristic of pure borneol.

#### Powder X-ray diffraction

PXRD analysis provided key evidence for phase identification. As shown in Fig. 3B, pure BRE displayed characteristic Bragg reflections at 2*θ* values of 7.9°, 10.2°, 14.3°, 16.1°, 25.9°, 26.8°, and 28.9°. BO showed intense peaks at 7.6° and 15.3°. The PM pattern was a simple summation of these peaks, indicating no interaction between the components. In contrast, the BRE-BO cocrystal pattern exhibited a distinct diffraction topology. Notably, new diagnostic peaks emerged in the 2*θ* range of 11°–13° (highlighted by the shaded band) and 14°–16.5°, which were absent in the physical mixture. This unique crystalline fingerprint, together with the thermal behavior (DSC), spectroscopic shifts (FTIR), and changes in local molecular environment (ssNMR) described above, provides compelling evidence that BRE and BO co-crystallize as a single-phase drug–drug cocrystal, rather than existing as a eutectic mixture or segregated domains.

#### Vibrational spectroscopy (FTIR)

FTIR analysis (Fig. 3C) was employed to probe the hydrogen bonding synthons. Pure BRE showed characteristic bands at 3519 cm⁻^1^ (O–H stretch), 1608 cm⁻^1^ (C = O stretch), and 1046 cm⁻^1^ (C–O–C stretch). In the cocrystal, significant spectral shifts were observed: the O–H and C = O bands shifted to lower wavenumbers, indicative of strong intermolecular hydrogen bonding (heterosynthons) between the hydroxyl donors of BRE/BO and carbonyl acceptors. The presence of C–H···π interactions between the aromatic rings of BRE and the bicyclic framework of BO suggests a geometrically complementary fit between the flavonoid and terpenoid scaffolds. Furthermore, the blueshift in the C–O–C stretching band of BRE (1046→1091 cm⁻^1^) suggests stabilization of the glycosidic bond via interaction with the steric terpene ring of BO.

#### Molecular environment analysis

Proton (^1^H) ssNMR (Fig. 3D) provided insight into the local molecular environment. Pure BO displayed a sharp methyl signal near 0 ppm, while BRE showed broad aromatic signals. The PM spectrum exhibited no discernible changes in chemical shifts relative to the individual components. In contrast, the cocrystal spectrum revealed significant broadening and upfield shifts of the signals near 0 ppm (associated with the methyl groups of BO), along with distinct new splitting patterns in the 0 to 10 ppm region. These perturbations confirm that the BO molecules reside in a chemically distinct environment within the cocrystal lattice, constrained by specific flavonoid–terpenoid interactions [21,22].

Collectively, the multimodal solid-state characterization provides converging evidence for the formation of a distinct BRE-BO drug–drug cocrystal phase. Thermal analysis confirms the incorporation of BO into a new crystalline environment with altered lattice energetics. Diffraction data establish the existence of a unique crystallographic phase. Vibrational and nuclear magnetic resonance spectroscopies reveal the molecular basis of this assembly: specific hydrogen bonding and van der Waals interactions between the flavonoid pharmacophore of BRE and the terpenoid framework of BO. This supramolecular organization not only stabilizes the volatile borneol component but also creates a crystalline matrix poised for synchronized dissolution—a prerequisite for achieving natural product synergy in vivo.

### Theoretical insights via DFT calculations

#### Energetics and stability

To validate the experimental findings, DFT calculations were performed. The single-point energy calculation yielded a binding energy (*ΔE*_bind_) of − 87.23 kcal/mol for the BRE-BO complex (Complex: − 1,368,258.92; BRE: − 1,075,100.64; BO: −293,071.05 kcal/mol). This substantial negative value indicates a highly exergonic formation process, suggesting that the cocrystal assembly is thermodynamically spontaneous and stable. This substantial binding energy, exceeding typical hydrogen bond strengths, reflects the synergistic contribution of multiple non-covalent contacts and explains the physical stability of the cocrystal under ambient storage conditions.

#### Energy decomposition analysis (EDA)

**Fig. 4:**
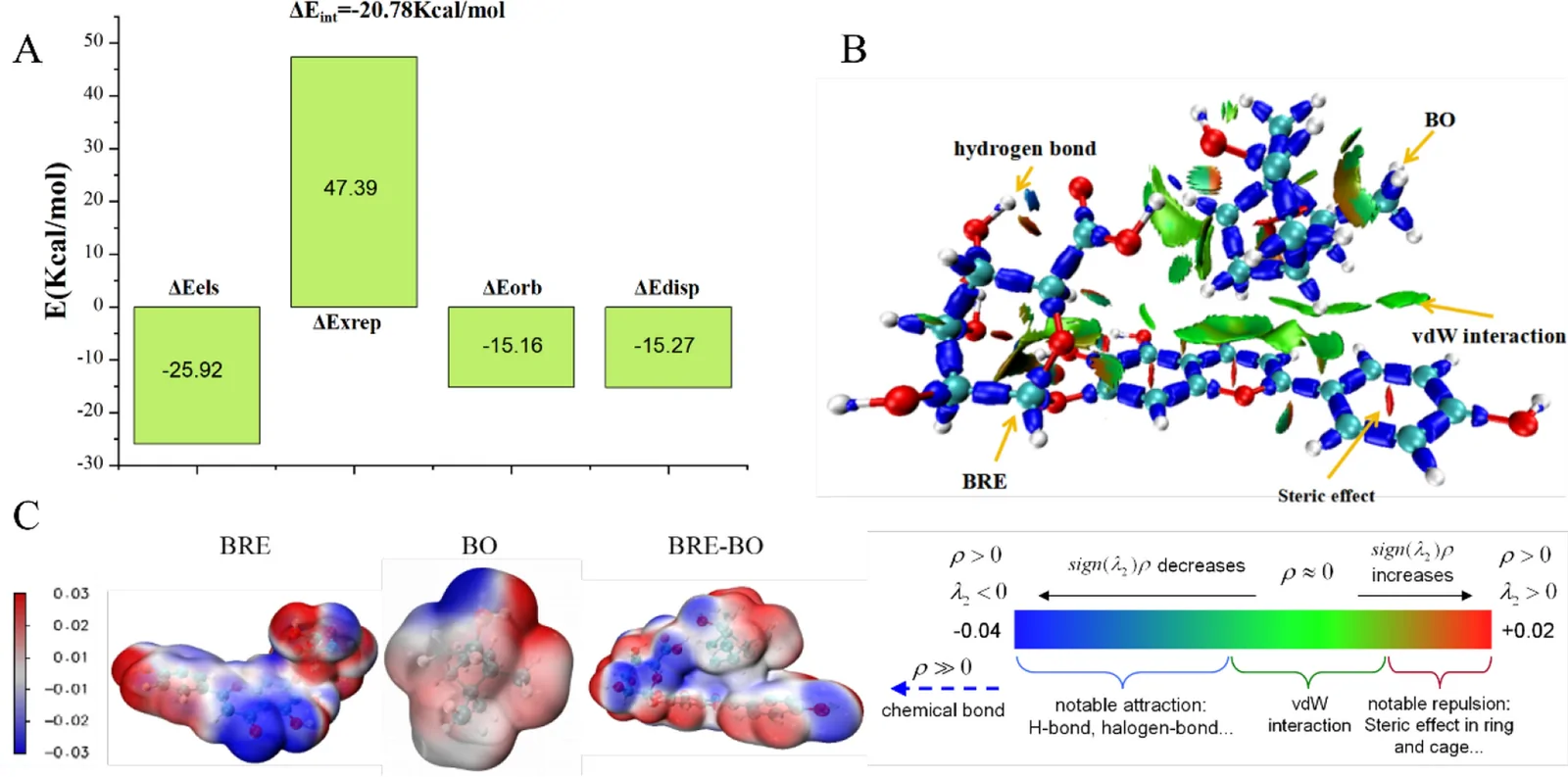
Computational analysis of the BRE-BO complex: **A** Energy decomposition analysis (EDA) histogram, **B** Visualization of non-covalent interactions via the interaction region indicator (IRI) method; **C** Molecular electrostatic potential (MEP) mapping

The nature of the intermolecular forces was dissected using the sobEDA method (Fig. 4A) [23]. For the most energetically favorable conformer (38.34% population at 300 K), the total interaction energy (*ΔE*_int_) was − 20.78 kcal/mol (Fig. 4A). The decomposition revealed that electrostatic interactions (ΔE_els_ = − 25.92 kcal/mol) constituted the dominant driving force, followed by dispersion forces (ΔE_disp_ = − 15.27 kcal/mol) and orbital interactions (ΔE_orb_ = − 15.16 kcal/mol). Although the exchange-repulsion term was significant (ΔE_xrep_ = 47.39 kcal/mol), it was effectively counterbalanced by the attractive forces. The dominance of electrostatic interactions is consistent with the charge complementarity expected between the electron-rich flavonoid core and the polarizable terpenoid hydroxyl group.

Visualizing these interactions via IRI analysis (Fig. 4B) revealed extensive green isosurfaces at the interface between the BRE hydroxyl/carboxyl groups and the BO hydroxyl group, characteristic of strong van der Waals and hydrogen bonding interactions.

#### Electrostatic potential and frontier orbitals

MEP mapping (Fig. 4C) highlighted the charge complementarity driving the assembly. The electron-deficient blue regions (H-bond donors) of BO aligned favorably with the electron-rich red regions (H-bond acceptors, C = O) of BRE, facilitating electrostatic anchoring [24].

**Fig. 5:**
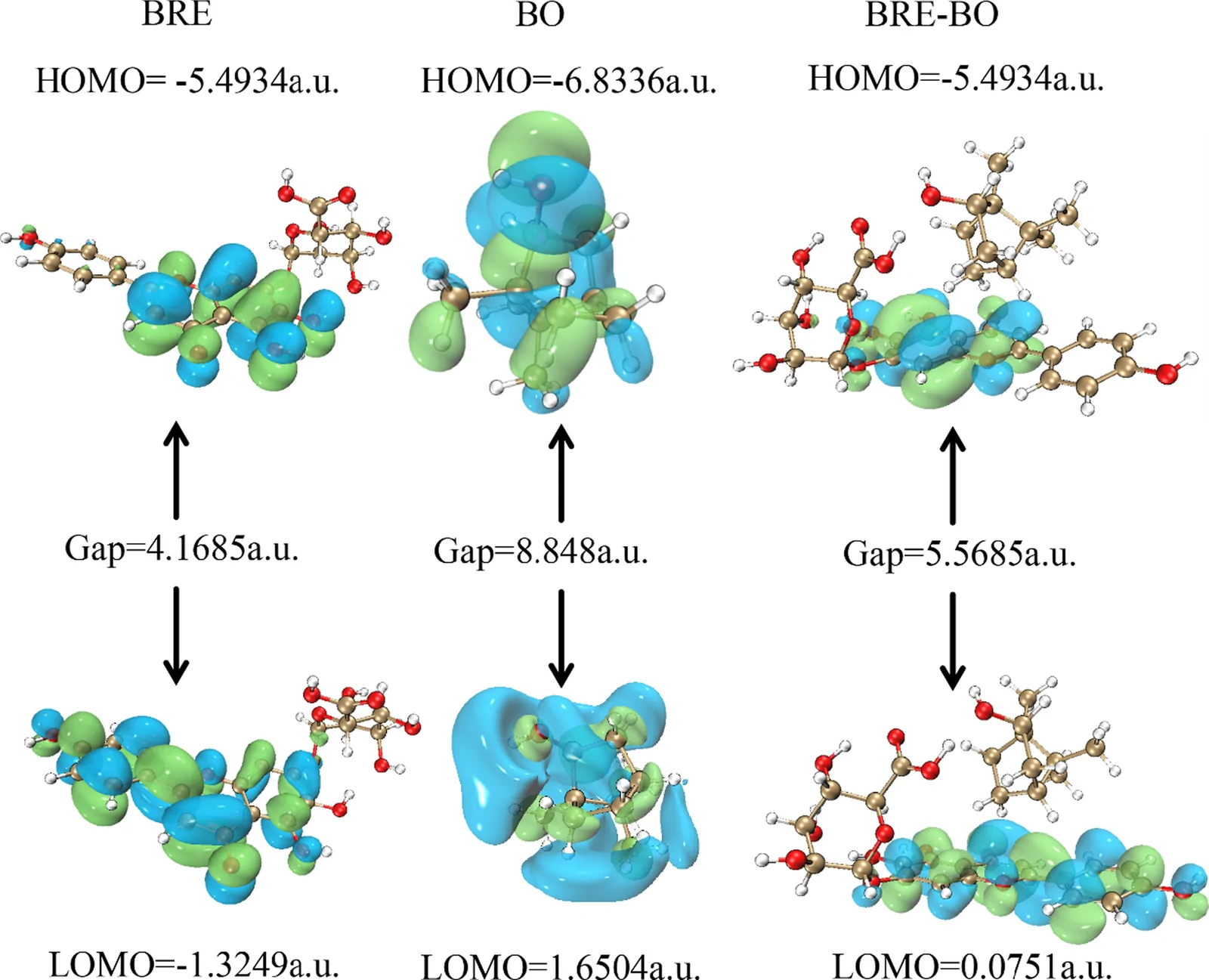
Frontier Molecular Orbital (FMO) analysis: Visualization of HOMO and LUMO surfaces and energy gaps for BRE, BO, and the BRE-BO complex

FMO analysis (Fig. 5) elucidated the electronic stability of the system. The HOMO of BRE was localized on the electron-rich γ-pyranone and benzene rings, whereas BO displayed a continuous orbital distribution that may facilitate proton transfer channels. The energy gap (ΔE_gap_) of the complex (5.5685 a.u.) was intermediate between those of BRE (4.1685 a.u.) and BO (8.848 a.u.) [25]. The HOMO–LUMO analysis indicates that cocrystallization does not significantly alter the intrinsic electronic properties of the scutellarin pharmacophore, suggesting that the therapeutic activity of BRE is preserved in the cocrystal form. The electronic reorganization upon complexation suggests that the cocrystal possesses a balanced reactivity profile, enhancing the chemical stability of BRE while maintaining the necessary reactivity for biological dissolution.

Taken together, the computational analyses substantiate the experimental findings by revealing the energetic and structural basis for BRE-BO cocrystal formation. The calculations demonstrate that the natural flavonoid and terpenoid components engage in a geometrically and electrostatically complementary assembly, driven predominantly by hydrogen bonding and dispersion interactions, without compromising the electronic integrity of the bioactive scutellarin moiety.

### In vitro drug release and kinetic analysis

The dissolution behaviors of BRE and BO from the pure drug, physical mixture, and cocrystal were evaluated in phosphate-buffered saline (PBS, pH 6.8).

**Fig. 6:**
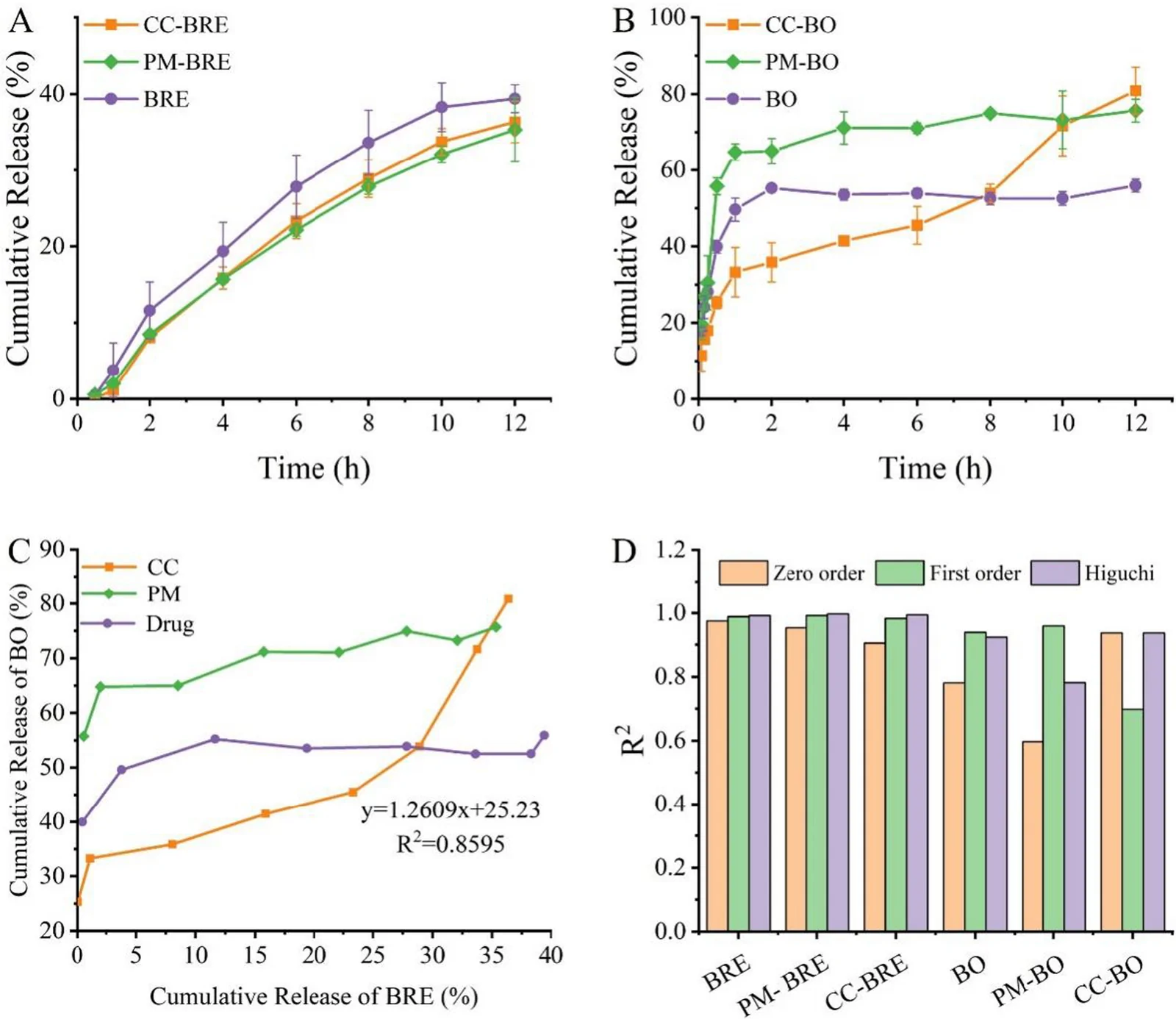
In vitro release dynamics and kinetic modeling: **A** Cumulative release of BRE and **B** BO from bulk drugs, PM, and CC; **C** Correlation of BRE vs. BO release fractions in the cocrystal; **D** Coefficients of determination (*R*^2^) for various kinetic models

As shown in Fig. 6A, the release profiles of BRE were broadly comparable across all three formulations, with the bulk drug exhibiting a marginally higher cumulative release than the PM and CC. This observation underscores the intrinsic solubility limitations of the flavonoid skeleton. However, the release behavior of BO (Fig. 6B) varied significantly among the groups. Bulk BO displayed a rapid release profile, reaching a plateau (~ 55%) within 2 h. The physical mixture exhibited a pronounced “burst effect”, releasing approximately 65% of BO within the first hour. The burst release likely arises from the rapid dissolution of surface dissolution of surface-borneol particles that are not protected within a crystalline matrix.

In contrast, the BRE-BO cocrystal demonstrated a unique sustained-release profile for the volatile terpenoid component. Following an initial release of ~ 25% at 30 min, the cumulative release of BO increased gradually, reaching nearly 80% by 12 h. This sustained behavior suggests that the incorporation of BO into the cocrystal lattice effectively mitigates its rapid sublimation and dissolution, with release kinetics controlled by the gradual dissociation of the supramolecular structure [26,27]. In contrast to the physical mixture, the cocrystal lattice imposes a kinetic barrier to BO release, requiring the dissociation of hydrogen-bonded BRE-BO units prior to solubilization.

Correlation analysis of the cumulative release fractions of BRE versus BO (Fig. 6C) revealed a superior synchronization in the cocrystal formulation (*R*^2^ = 0.86) compared to the physical mixture. The curve indicates a biphasic release pattern: a rapid initial phase followed by a synchronized sustained release. This synchronized release profile is a critical prerequisite for achieving natural product synergy in vivo, as it ensures that both the therapeutic flavonoid and the permeation-enhancing terpenoid are presented simultaneously to the intestinal epithelium. The biphasic behavior suggests that the co-evaporation process successfully embedded both components within a unified crystalline lattice, thereby preventing the discordant, independent dissolution observed for the physical mixture [28,29].

Kinetic modeling (Fig. 6D) indicated that BRE release from all formulations was best described by the Higuchi model (*R*^2^ > 0.99), consistent with diffusion-controlled release from a matrix. For BO, the bulk and PM formulations followed first-order kinetics, whereas the cocrystal release fitted best to the Higuchi model (*R*^2^ > 0.94). Further analysis using the Ritger–Peppas equation yielded a diffusion exponent (n) of 1.49 for BRE in the cocrystal, indicative of Super Case-II transport (relaxation-controlled), while BO exhibited Fickian diffusion (n = 0.38) [30]. The Super Case-II transport observed for BRE suggests that drug release is governed predominantly by lattice erosion or polymer relaxation rather than simple diffusion. This mechanism is advantageous for sustained delivery, as it provides a more predictable and prolonged release profile compared to diffusion-controlled systems. The modified and synchronized release kinetics observed in vitro provide a mechanistic basis for anticipating altered pharmacokinetic behavior in vivo.

### Storage stability assessment

**Fig. 7:**
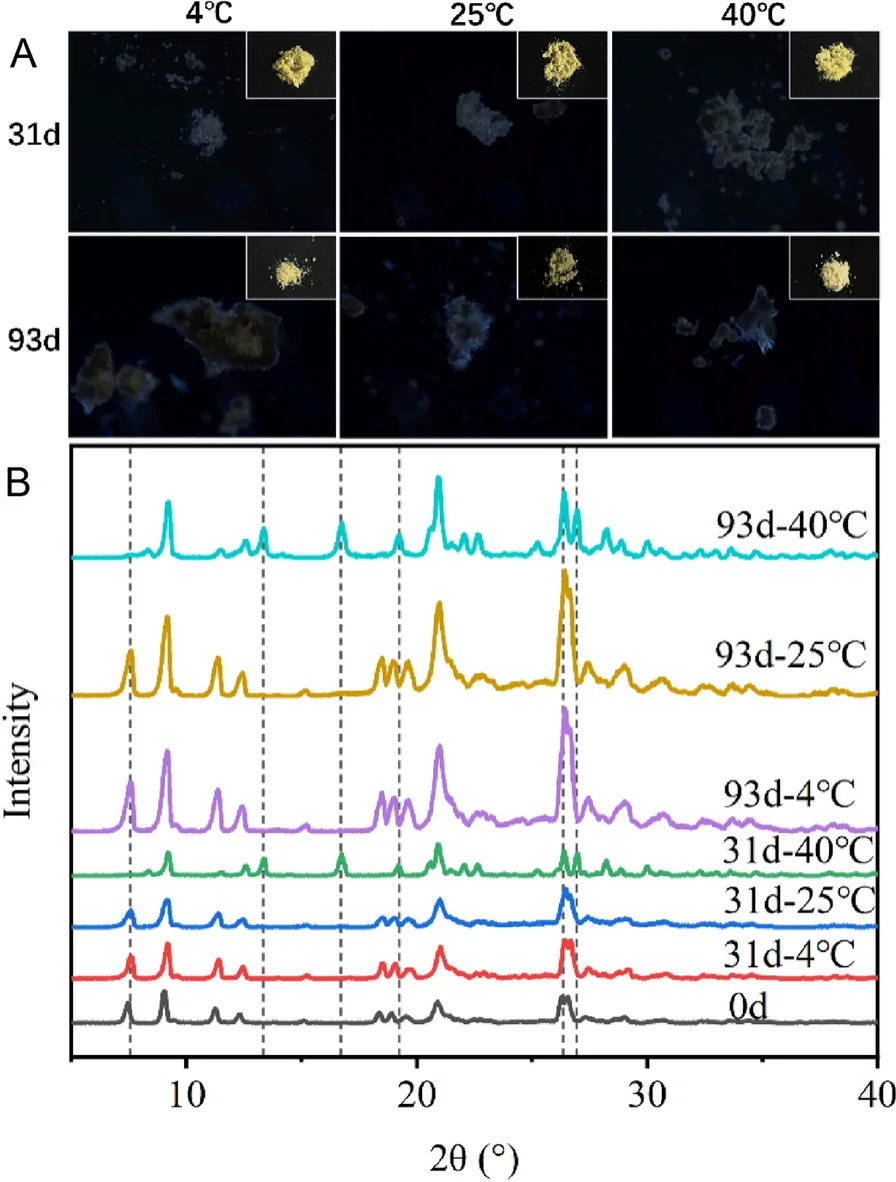
Stability evaluation of BRE-BO cocrystals: **A** Macroscopic appearance; **B** PLM micrographs exhibiting birefringence; **C** PXRD patterns after storage at 4 °C, 25 °C, and 40 °C

The physical stability of the BRE-BO cocrystals was monitored under stress conditions over a 3-month period (Fig. 7).

Under refrigerated (4 °C) and ambient (25 °C, 65% RH) conditions, the cocrystals maintained their macroscopic appearance (pale yellow powder) and crystalline integrity. PXRD patterns at 93 days remained identical to those of freshly prepared samples (0 days), confirming phase stability under these storage conditions. However, accelerated aging at 40 °C induced noticeable changes. PLM imaging revealed altered birefringence patterns after 3 months of storage at elevated temperature. Correspondingly, the PXRD diffractogram of the cocrystal stored at 40 °C for 3 months exhibited notable deviations from the freshly prepared sample. Specifically, the characteristic peaks of freshly prepared cocrystals emerged at *2θ* = 13.4° and 16.7°, while the characteristic peak at *2θ* = 7.6° was markedly attenuated. In addition, the split peaks originally observed near *2θ* = 19.2° merged into a single, weakened reflection, and the peak at *2θ* = 26.5° underwent splitting into two distinct peaks at *2θ* = 26.4° and 27.0°. These spectral changes indicate partial phase transformation or lattice rearrangement under thermal stress.

These findings suggest that while the cocrystal is robust at standard storage temperatures, prolonged exposure to thermal stress (40 °C) may trigger partial phase dissociation or recrystallization [31]. The thermal stress at elevated temperature may provide sufficient energy to overcome the intermolecular interactions maintaining lattice integrity. Consequently, storage at cool or ambient temperatures is recommended to ensure long-term stability of the BRE-BO cocrystal formulation.

### In vivo pharmacokinetic evaluation

**Fig. 8:**
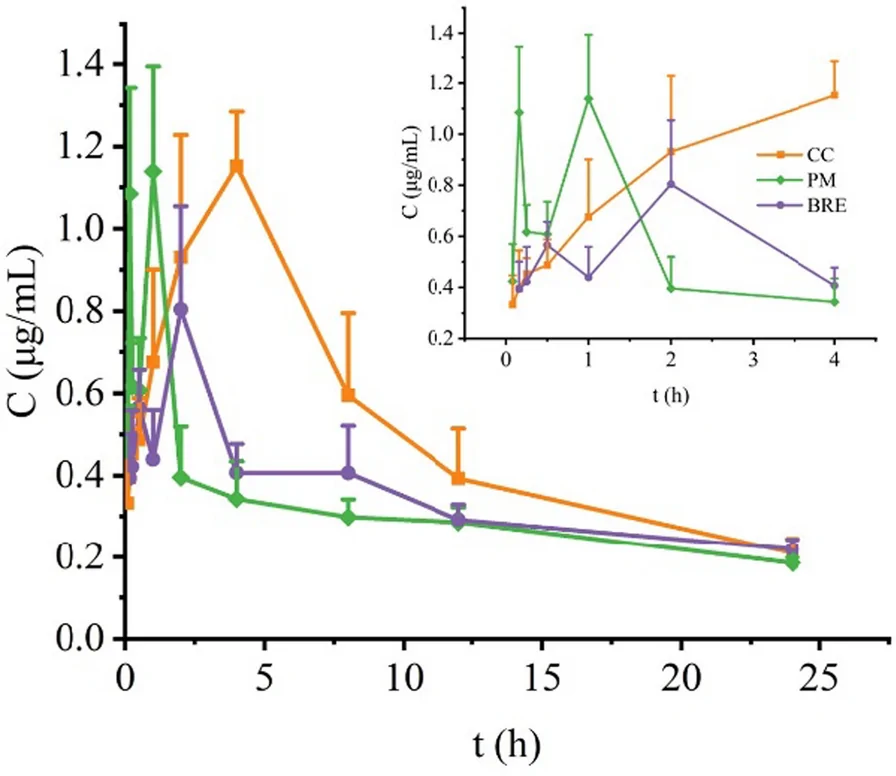
Mean plasma concentration-time profiles of BRE following oral administration of bulk BRE, physical mixture (PM), and BRE-BO cocrystal (CC) in male SD rats (n = 6)

The in vivo pharmacokinetic study was designed to evaluate whether the synchronized release and co-delivery of BRE and BO from the drug–drug cocrystal could translate into tangible improvements in systemic exposure—the essence of natural product synergy. The plasma concentration–time profiles and key pharmacokinetic parameters are presented in Fig. 8 and Table 1, respectively.

**Table 1:** Pharmacokinetic parameters of BRE following oral administration of various formulations in male SD rats (mean ± SD, n = 6)

Formulations	Cocrystal	Physical mixture	Bulk BRE
AUC_(0-t)_, mg/L·h	12.473 ± 1.534^*#^	7.017 ± 1.458	7.797 ± 0.719
AUC_(0-∞)_, mg/L·h	14.898 ± 2.048	12.973 ± 5.172	12.49 ± 3.663
MRT_(0-t)_, h	8.417 ± 0.808	8.417 ± 2.168	9.018 ± 2.028
MRT_(0-∞)_, h	13.863 ± 4.693	30.965 ± 21.041	25.375 ± 10.814
t_1/2_, h	8.481 ± 4.225	22.242 ± 16.017	16.293 ± 9.734
T_max_, h	3.333 ± 1.033^#^	0.496 ± 0.46	2.333 ± 0.816
C_max_, mg/L	1.188 ± 0.17^*^	1.198 ± 0.275	0.804 ± 0.273

^*^p < 0.05, compared to bulk BRE

^#^*p* < 0.05, compared to physical mixture

The cocrystal formulation elicited a substantial improvement in the systemic exposure of BRE. The area under the curve (*AUC*_0-t_) for the cocrystal was increased by approximately 1.6-fold compared to bulk BRE (p<0.05) and 1.8-fold compared to the physical mixture (p<0.05) [32]. This enhancement in *AUC*_0-t_ represents a clinically meaningful improvement, as it may allow for dose reduction while maintaining therapeutic efficacy, potentially mitigating dose-related adverse effects associated with long-term flavonoid therapy.

Furthermore, the cocrystal significantly delayed the time to peak concentration (*T*_max_), extending to 3.33 h compared to 0.50 h for the physical mixture and 2.3 h for bulk BRE. The prolonged *T*_max_ observed for the cocrystal is consistent with the sustained release kinetics observed in vitro and suggests that the gradual dissociation of the cocrystal lattice in vivo provides a rate-limiting step for drug absorption.

This shift, combined with an elevated peak plasma concentration (*C*_max_) for cocrystal compared to that for bulk BRE, is characteristic of a sustained-release formulation that avoids the sharp “peak-and-trough” fluctuations seen in the physical mixture.

Interestingly, the calculated elimination half-life (*t*_1/2_) of BRE in the cocrystal group (8.5 h) was notably shorter than that of the physical mixture (22.2 h) and bulk BRE (16.3 h). While a longer *t*_1/2_ in the mixture might superficially imply extended residence, the significantly higher AUC in the cocrystal group indicates that the primary driver of enhanced systemic exposure is improved absorption rather than reduced elimination. The shorter *t*_1/2_ observed with the cocrystal may reflect more efficient clearance of the absorbed fraction, a phenomenon that is not detrimental when accompanied by substantially enhanced overall exposure.

Notably, the plasma concentration–time profiles of bulk BRE and the physical mixture exhibited a bimodal pattern (double-peak phenomenon), which is characteristic of flavonoids undergoing enterohepatic circulation [33]. This erratic fluctuation was effectively dampened in the cocrystal formulation, which displayed a smoother and more predictable absorption phase. The attenuation of the double-peak phenomenon in the cocrystal group suggests that synchronized co-delivery with borneol may modulate enterohepatic recirculation, possibly through altered glucuronidation kinetics or biliary excretion pathways.

The superior pharmacokinetic performance of the BRE-BO cocrystal can be rationalized by the convergence of physicochemical and biological synergies inherent to this drug–drug cocrystal design. From a physicochemical perspective, cocrystallization synchronizes the release of the two natural products, ensuring that the permeation enhancer (BO) is available at the absorption site concurrently with the therapeutic agent (BRE). This temporal coordination is absent in the physical mixture, where the rapid dissolution and absorption of BO precedes that of BRE, resulting in diminished biopharmaceutical enhancement. From a biological perspective, the well-documented activities of borneol—including reversible modulation of epithelial tight junctions to enhance paracellular permeability, inhibition of P-glycoprotein-mediated efflux, and attenuation of cytochrome P450-mediated first-pass metabolism—collectively facilitate the intestinal absorption and systemic preservation of scutellarin [13].

These findings validate the central hypothesis of this study: that drug–drug cocrystallization can harness natural product synergy to overcome the delivery barriers of poorly bioavailable flavonoids. The BRE-BO cocrystal strategy not only enhances oral bioavailability but also stabilizes plasma levels, presenting a promising excipient-free platform for the development of fixed-dose combinations derived from natural products [34].

## Conclusions

This study demonstrates a supramolecular strategy for optimizing the oral delivery of natural flavonoids through the rational engineering of a BRE-BO pharmaceutical cocrystal. By incorporating borneol as a dual-function agent—acting simultaneously as a crystal lattice stabilizer and a biological permeation enhancer—we achieved a synchronized and sustained release profile for both the active ingredient and the coformer. This structural integration translated directly into tangible pharmacokinetic benefits, yielding a 1.6-fold improvement in the oral bioavailability of breviscapine, characterized by a delayed *T*_max_ and enhanced *C*_max_ relative to the native extract and physical mixture.

The BRE-BOR cocrystal provides a proof-of-concept for the development of excipient-free, crystalline fixed-dose combinations derived from natural products. This drug–drug cocrystal approach reconciles traditional herbal compatibility principles with modern crystal engineering and provides a promising strategy for addressing the biopharmaceutical challenges of poorly bioavailable flavonoids. Future investigations will focus on pharmacodynamic evaluation, safety assessment, and clinical translation of this cocrystal system, along with a more detailed mechanistic elucidation of how borneol co-delivery modulates metabolic enzymes and transporter proteins in the intestinal epithelium.

## Materials and methods

### Materials and animals

Breviscapine (BRE; scutellarin content > 95%) was purchased from Jingzhu Biotechnology Co., Ltd. (Nanjing, China). Borneol (BO; purity > 98%) was supplied by Yuanye Biotech Co. (Shanghai, China). Analytical grade methanol and dimethyl sulfoxide (DMSO) were procured from Zhiyuan Chemical Reagent Co., Ltd. (Tianjin, China). High-performance liquid chromatography (HPLC)-grade MeOH and all other reagents were used as received without further purification. Ultrapure water was generated using a Sartorius Arium^®^ Comfort II system (Göttingen, Germany) and used throughout all experiments.

Male Sprague–Dawley (SD) rats (7–8 weeks old, 200–250 g) were sourced from Henan Scribe Biotechnology Co., Ltd. (License No. SCXK(YU)2020-0005) and housed in a specific pathogen-free environment under standard conditions (12 h light/dark cycle, 22 ± 2 °C, 50 ± 10% humidity). Animals were provided ad libitum access to standard chow and water. All animal procedures were conducted in accordance with the guidelines and approved by the Animal Experimental Committee of Yunnan University of Chinese Medicine (YNUTCM-XMSS-G-20250014).

### Preparation of BRE-BO cocrystals

The BRE-BO cocrystals were prepared using a solvent evaporation technique, with minor modifications to previously reported protocols [35]. Briefly, BRE and BO were dissolved in methanol at a 1:1 molar ratio to achieve a final solute concentration of 10.0% (w/v). The resulting clear solution was subjected to solvent removal under reduced pressure at 40 °C using a rotary vacuum evaporator (RE212B, Yamato, Japan). The concentrated residue was subsequently dried at room temperature for 72 h to ensure complete solvent elimination. The final solid product was collected, gently ground, and stored in hermetically sealed vials within a desiccator for further characterization.

### Morphology characterization

The surface morphology and optical properties of BRE, BO, their physical mixture (PM), and the BRE-BO cocrystals were examined using polarized light microscopy (PLM) and scanning electron microscopy (SEM). Birefringence patterns were visualized using an MXP-260 PLM (Precise, China) equipped with NScope 2.0 image acquisition software. For detailed microstructural analysis, samples were mounted on conductive carbon tape, sputter-coated with a thin gold layer, and imaged using a Phenom Pro SEM (Phenom-World, Eindhoven, Netherlands) at an accelerating voltage of 10 kV.

### Physicochemical characterization

#### Powder X-ray diffraction (PXRD)

Crystallographic patterns were recorded on a D8 Advance diffractometer (Bruker, Karlsruhe, Germany). Analysis was performed over a 2*θ* range of 5°–40° with a step size of 0.02° and a scanning speed of 5°/min. The resulting diffraction patterns were analyzed to identify unique crystalline phases and to distinguish the cocrystal structure from those of the individual components.

#### Differential scanning calorimetry (DSC)

Thermal behavior was evaluated using a Setsys Evo 16 DSC instrument (Setaram, Caluire, France). Approximately 5–10 mg of each powder sample was accurately weighed into aluminum pans and hermetically sealed. Samples were heated from 50 °C to 300 °C at a scan rate of 10 °C/min under a nitrogen atmosphere. Melting points and enthalpy of fusion (ΔH) were determined by integration of the endothermic peaks using the instrument software.

#### Fourier-transform infrared spectroscopy (FTIR)

Vibrational spectra were acquired using a Ruijie Great20 spectrophotometer (Tianjin, China). Samples were prepared by grinding with spectroscopic-grade potassium bromide and compressing the mixture into translucent pellets. Spectra were recorded over the wavenumber range of 4000–400 cm⁻^1^ at a resolution of 2 cm⁻^1^ to detect shifts in functional group vibrations indicative of intermolecular interactions.

#### Solid-state nuclear magnetic resonance (ssNMR)

Proton (^1^H) solid-state NMR spectra were acquired using an Avance DRX400 spectrometer (Bruker Biospin GmbH, Germany) operating at 400 MHz. To mitigate interference from adsorbed moisture, samples were finely ground and vacuum-dried prior to analysis. A single-pulse sequence was employed to probe the local magnetic environment of BRE and BO protons within the cocrystal lattice, thereby providing evidence of supramolecular assembly.

### Computational studies and DFT calculation

Density functional theory (DFT) calculations were performed to elucidate the atomic-level mechanism of cocrystal formation. The initial molecular geometries of BRE and BO were retrieved from the PubChem database. All quantum chemical calculations were carried out using the ORCA software package [36]. Geometry optimizations and vibrational frequency analyses were conducted at the B3LYP-D3 (BJ)/6-311G* level of theory [37]; the absence of imaginary frequencies confirmed that the optimized geometries corresponded to true local minima. Refined single-point energies were calculated at the wB97XD/6-311G** level to ensure energetic accuracy [38].

For the BRE-BO supramolecular complex, a rigorous conformational search was implemented. One hundred initial dimer structures were generated using Molclus software [39] based on monomers optimized at the B3LYP-D3(BJ)/6-31G* level. These configurations underwent preliminary optimization, and the 20 lowest-energy conformers were retained. Subsequent optimization at the B3LYP-D3 (BJ)/6-311G* level identified the 10 most stable conformers. Energy decomposition analysis (EDA) and single-point energy calculations were performed at the wB97XD/6-311G** level. The population distribution of each conformer was determined via Boltzmann distribution analysis at 300 K.

To visualize non-covalent interactions, molecular electrostatic potential (MEP) surfaces were mapped, and the interaction region indicator (IRI) method [40] was applied. Frontier molecular orbitals (HOMO/LUMO) were analyzed to assess electronic stability. The sobEDA method [23] was employed to decompose the total interaction energy into electrostatic, exchange-repulsion, orbital interaction, and dispersion components. All graphical visualizations were generated using Multiwfn [41,42] and VMD [43] software.

### In vitro drug release kinetics

In vitro drug release profiles were evaluated using the dialysis bag method under sink conditions. Samples were enclosed in dialysis membranes (MWCO 3000 Da) and immersed in 100 mL of phosphate-buffered saline (PBS, pH 6.8) maintained at 37 ± 0.5 °C. The dissolution medium was agitated at 300 rpm using a paddle apparatus. At predetermined intervals, 2 mL aliquots were withdrawn and immediately replaced with an equal volume of fresh pre-warmed medium. All samples were filtered through a 0.22 μm membrane prior to analysis.

BRE was quantified using a Shimadzu LC-30A HPLC system (Kyoto, Japan) equipped with a Cosmosil 5C18-AR-II column (4.6 mm × 250 mm, 5 µm). The mobile phase consisted of acetonitrile and 0.1% phosphoric acid (21:79, v/v) delivered at a flow rate of 1 mL/min, with UV detection at 335 nm.

BO concentration was determined via gas chromatography (GC) using a PEG-20 M capillary column (30 m × 0.25 mm × 0.25 µm) with a flame ionization detector (FID). The injector and detector temperatures were maintained at 240 °C. Nitrogen was used as the carrier gas. The column temperature was programmed from 80 °C to 180 °C at a ramp rate of 10 °C/min.

The cumulative release percentage $\left(Q\right)$ was calculated using the following equation:

$$Q = C_{n} V + \frac{{\sum C_{i} V_{i} }}{{Q_{0} }} \times 100$$

where *V* is the total volume of the release medium, *Q*_0_ is the initial drug loading, *C*_n_ is the drug concentration at time *n*, *C*_i_ and *V*_i_ represent the concentration and volume of the sample withdrawn at time *i*, respectively.

Release data were fitted to zero-order, first-order, Higuchi, and Ritger-Peppas models to elucidate the release mechanism. The model with the the highest correlation coefficient (*R*^2^) was considered the best fit.

### Physical stability assessment

To evaluate storage stability, BRE-BO cocrystals were stored in sealed vials under three distinct conditions: 4 °C, 25 °C, and 40 °C, all maintained at 65% relative humidity (RH). At predetermined time points, samples were inspected for macroscopic changes in appearance. Microscopic integrity and crystallinity were monitored using PLM and PXRD, respectively. Diffractograms and birefringence patterns were compared against those of freshly prepared cocrystals to detect any phase transformation or degradation.

### Pharmacokinetic evaluation

The oral pharmacokinetic performance of the BRE-BO cocrystal was compared with that of the physical mixture and pure BRE in male SD rats. Animals were randomly assigned to three groups (n = 6 per group) and administered a single oral dose equivalent to 200 mg/kg of BRE via intragastric gavage. The BRE-BO cocrystal group and the physical mixture group both utilized a BRE:BO molar ratio of 1:1. For all three groups, the administered dose was normalized to deliver an equivalent BRE dose of 200 mg/kg body weight. Blood samples (approximately 0.5 mL) were collected from the retro-orbital plexus at 0, 5, 15, 30 min, and 1, 2, 4, 8, 12, and 24 h post-administration.

Plasma was harvested by centrifugation at 4000 rpm for 10 min at 4 °C. A 100 μL aliquot was spiked with 20 μL of 8 mol/L phosphoric acid and 20 μL of internal standard (rutin, 8 μg/mL in methanol). Protein precipitation was induced by the addition of 500 μL acetonitrile/ethyl acetate mixture (3:1, v/v). Following vortex-mixing for 5 min and high-speed centrifugation (12,000 rpm, 10 min), the supernatant was collected and evaporated to dryness under a gentle stream of nitrogen. The residue was reconstituted in 200 μL of methanol, centrifuged again, and a 10 μL aliquot of the resulting supernatant was injected into the HPLC system for analysis.

Pharmacokinetic parameters were calculated using DAS 2.0 software via non-compartmental analysis. The analytical method was validated for linearity (0.25–8 μg/mL, *R*^2^ = 0.999), precision (intra- and inter-day RSD < 10%), extraction recovery (88.99–93.42%), and matrix effects (92.51–102.70%).

### Statistical analysis

All quantitative data are expressed as mean ± standard deviation (SD). Statistical analyses were performed using SPSS software (version 27; IBM Corp., Armonk, NY, USA). Differences among groups were assessed by one-way analysis of variance (ANOVA) followed by appropriate post hoc tests for multiple comparisons. A *p* value < 0.05 was considered statistically significant.

## Data Availability

The data that support the findings of this study are available from the corresponding author upon reasonable request.
